# Screening for Plasminogen Mutations in Hereditary Angioedema Patients

**DOI:** 10.3390/genes12030402

**Published:** 2021-03-11

**Authors:** Henriette Farkas, Anna Dóczy, Edina Szabó, Lilian Varga, Dorottya Csuka

**Affiliations:** 1 Hungarian Angioedema Center of Reference and Excellence, Department of Internal Medicine and Haematology, Semmelweis University, H-1088 Budapest, Hungary; varga.lilian@med.semmelweis-univ.hu (L.V.); csuka.dorottya@med.semmelweis-univ.hu (D.C.); 2 Heart and Vascular Center, Semmelweis University, H-1122 Budapest, Hungary; doczy.anna@med.semmelweis-univ.hu; 3 MTA-SE Research Group of Immunology and Haematology, Research Laboratory, Department of Internal Medicine and Haematology, Hungarian Academy of Sciences and Semmelweis University, H-1088 Budapest, Hungary; szabo.edina@med.semmelweis-univ.hu

**Keywords:** hereditary angioedema, plasminogen, mutation, family screening, biorepository

## Abstract

Hereditary angioedema (HAE) is a rare disease belonging to the group of bradykinin-mediated angioedemas, characterized by recurring edematous episodes involving the subcutaneous and/or submucosal tissues. Most cases of HAE are caused by mutations in the *SERPING1* gene encoding C1-inhibitor (C1-INH-HAE); however, mutation analysis identified seven further types of HAE: HAE with Factor XII mutation (FXII-HAE), with plasminogen gene mutation (PLG-HAE), with angiopoietin-1 gene mutation (ANGPT1-HAE), with kininogen-1 gene mutation (KNG1-HAE), with a myoferlin gene mutation (MYOF-HAE), with a heparan sulfate-glucosamine 3-sulfotransferase 6 (*HS3ST6*) mutation, and hereditary angioedema of unknown origin (U-HAE). We sequenced DNA samples stored from 124 U-HAE patients in the biorepository for exon 9 of the *PLG* gene. One of the 124 subjects carried the mutation causing a lysine to glutamic acid amino acid exchange at position 330 (K330E). Later, the same *PLG* mutation was identified in the patient’s son. The introduction of new techniques into genetic testing has increased the number of genes identified. As shown by this study, a biorepository creates the means for the ex-post analysis of recently identified genes in stored DNA samples of the patients. This makes the diagnosis more accurate with the possibility of subsequent family screening and the introduction of appropriate therapy.

## 1. Introduction

Hereditary angioedema (HAE) is a rare but life-threatening disorder belonging to the group of bradykinin-mediated angioedemas. It is characterized by recurring edematous episodes involving the subcutaneous or submucosal tissues. In the upper airways, mucosal edema may cause suffocation, while in the gut, the swelling of the intestinal mucosa may elicit symptoms resembling those of an acute “abdominal catastrophe”. Being a rare disorder, it is little known among medical professionals and its identification requires special laboratory background. Thus, its diagnosis is often belated and this imposes a substantial disease burden on the patients. Complement testing can distinguish with certainty two major groups of HAE—that is, HAE with C1-INH deficiency (C1-INH-HAE), and HAE with normal C1-INH function (nC1-INH-HAE) [1]. In C1-INH-HAE, mutations in the *SERPING1* gene determine two different subtypes: In type I C1-INH-HAE, both the antigenic C1-INH concentration and the functional C1-INH activity are low, as no protein is generated from the mutant allele. On the contrary, a functionally inactive mutant C1-INH is detectable in type II C1-INH-HAE, that is characterized by normal or even elevated serum C1-INH level, along with decreased functional C1-INH activity [1]. In nC1-INH-HAE, genetic testing is necessary to establish the diagnosis. Currently, mutation analysis can distinguish seven types of nC1-INH-HAE: HAE with Factor XII mutation (FXII-HAE) [2], HAE with plasminogen gene mutation (PLG-HAE) [3,4], HAE with angiopoietin-1 gene mutation (ANGPT1-HAE) [5], HAE with kininogen-1 gene mutation (KNG1-HAE) [6], HAE with a myoferlin gene mutation (MYOF-HAE) [7], HAE with a heparan sulfate-glucosamine 3-sulfotransferase 6 (*HS3ST6*) mutation [8], and hereditary angioedema of unknown origin (U-HAE). Concerning the latter, no diagnostic laboratory test exists. Therefore, the diagnosis is based on the exclusion of C1-INH deficiency and nC1-INH-HAE with a known genetic background, as well as on the clinical symptoms, the positive family history (one or more family members also affected with AE), and the inadequate response to conventional therapy (with antihistamines, corticosteroids and epinephrine). Remarkably, patients suffering from PLG-HAE are characterized by a later disease onset (in adulthood typically) and by edematous swellings localized to the tongue, larynx and the face [3,9]. Plasminogen, the precursor of plasmin, is produced in the liver and is secreted in extracellular fluids [10]. Native plasminogen includes the following parts: an N-terminal peptide, five kringle domains, and a protease domain in the C-terminal part. The generated protease, plasmin has a wide range of substrates: it can cleave fibrin, elements of the extracellular matrix (fibronectin, laminin, collagen type IV), and it can activate several matrix metalloprotease zymogens, as well as different growth factors, cytokines and chemokines [11].

In the current study, we study the incidence of the missense *PLG* mutation (p.Lys330Glu, K330E) described earlier [3,4] in DNA samples obtained from our U-HAE patients and stored in a biorepository.

## 2. Subjects and Methods

Between 2009 and 2019, we undertook targeted outpatient screening of approx. 5000 patients referred for U-HAE to the National Angioedema Reference Center. The clinical and laboratory data of the patients diagnosed with hereditary C1-INH deficiency or with nC1-INH-HAE were recorded in the National Angioedema Registry. From the blood samples drawn at the initial visit, (EDTA or citrated) plasma and serum samples (stored at −80 °C), as well as DNA obtained following DNA isolation were stored (at −20 °C) in the biorepository. Genomic DNA was extracted from peripheral blood according to the salting out procedure described by Miller et al. [12]. The study protocol was approved by the institutional review board of the Semmelweis University of Budapest, and informed consent was obtained from the participants in accordance with the Declaration of Helsinki. According to the diagnostic criteria, and because the analysis of the *SERPING1* and *F12* genes were negative for mutations, U-HAE was diagnosed in 124 patients (35 males and 89 females with a mean age of 41 years (min.: 9, max.: 94 years)) (previously, 53 unrelated U-HAE patients were screened for mutations in *PLG* and further genes [13] without overlap between the current and the previously published patient groups). Fifty-one of these 124 patients belonged to 16 families (DNA samples were unavailable from the family members of the remaining 73 subjects). In this population, the initial onset of angioedema (AE) symptoms usually occurred at the age of 30 years. Angioedema most commonly involved the face (20.8%) and the lips (21.3%). Additional locations included the lower and upper extremities (12.4%), the tongue (7.9%), the genitals (2.5%), and the upper airway mucosa (1%); abdominal attacks occurred in 6.4% of the subjects.

In 2019, we introduced the analysis of the plasminogen (*PLG*) gene in HAE (testing for mutations in the *ANGPT1* and *KNG1* genes is not yet available in our center). The DNA samples stored from the 124 patients in the biorepository were sequenced for exon 9 of the *PLG* gene by direct bidirectional DNA sequencing following PCR amplification, using the following forward and reverse primers: 5′-CTTAGTTTTAGTTACTGTAGGAACGCAGG-3′ and 5′-CAGGCTTTCTGACCACAATAGC-3′, as described previously by Bork et al. [3]. The PCR was initiated at 95 °C for 5 min, followed by denaturation at 94 °C for 15 s, annealing at 60 °C for 12 s, and extension at 72 °C for 30 s, for 35 cycles. The PCR products were sequenced using the BigDye Terminator v3.1 Cycle Sequencing Kit (Life Technologies Corporation, Carlsbad, CA 92008, USA) and analyzed in ABI 3730 DNA Analyzer (Life Technologies Corporation, Carlsbad, CA 92008, USA). Sequences were assembled to a reference sequence using the CLC Main Workbench 8.0.1. (QIAGEN, Aarhus, Denmark).

## 3. Results

One of the 124 subjects (Patient E385) carried the mutation causing Lys > Glu amino acid change at position 330.

**Figure 1:**
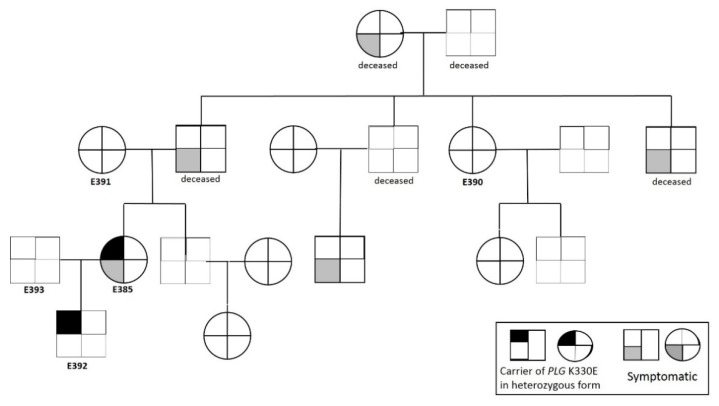
Family tree of the index case. Blood samples were available only from the subjects labelled with numbers (E385, E390, E391, E392, E393), they were screened for the *PLG* K330E mutation by Sanger-sequencing.

The 60-year-old index patient (E385) diagnosed with PLG-HAE (Figure 1) had a positive family history: Her father has had recurrent edema of the tongue and face. Moreover, edema of the face, larynx, and tongue occurred in her paternal grandmother, the sibling of her father, and the patient’s cousin. 

**Figure 2:**
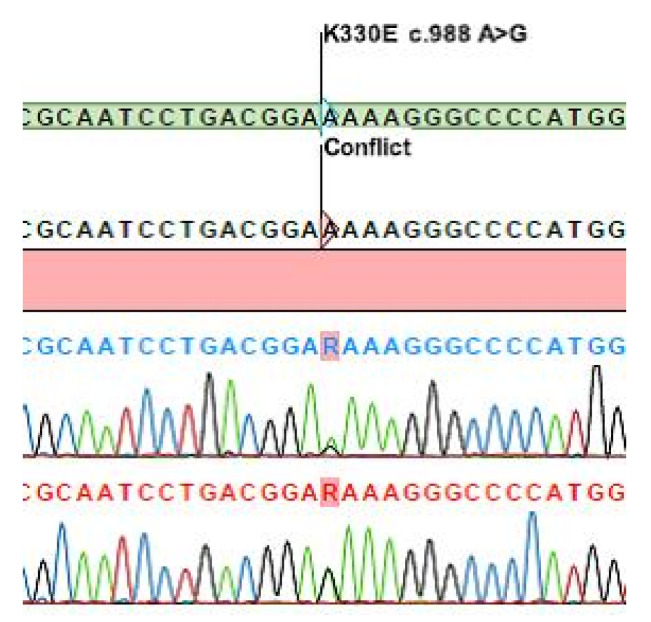
Sequence showing the identified *PLG* K330E mutation.

The patient sustained her initial AE attack (facial edema) at the age of 17 years; however, it was diagnosed as an allergic reaction. Then, she was symptom-free for the subsequent 20 years until she was diagnosed with hypertension at the age of forty, and perindopril treatment was introduced. An AE attack recurred in the form of tongue edema while she was taking the ACEI. ACEI-AAE was suspected and perindopril was replaced by an angiotensin II receptor blocker (telmisartan). However, as edema of the tongue and face recurred on three occasions, the patient was switched to a combination of rilmenidin (an imidazoline receptor agonist) and nebivolol (a β-blocker). This was to no avail as episodic tongue and face edema kept on recurring for a further four occasions. The patient received emergency care on every occasion: After treatment with antihistamines and corticosteroids, tongue edema resolved in 6 to 12 h; however, this took three to four days in the case of facial edema. The patient was referred to our Center for severe facial edema unresponsive to standard therapy. As complement testing excluded C1-INH-HAE and no mutations could be detected in the *F12* gene, the patient was diagnosed with U-HAE. A year later, during the testing of U-HAE samples from the repository, Sanger-sequencing identified *PLG* K330E mutation [3,4] (c.988A > G) (Figure 2), and the patient was transferred from the U-HAE group into the PLG-HAE group. 

Next, we performed a pedigree analysis. Her symptom-free son (Patient E392) was also shown to carry the same mutation in the *PLG* gene. The genetic screening of three available family members (Patients E390, E391, and E393) was negative; the remaining—possibly affected—family members could not be reached (Figure 1). The patient and her son have been enrolled in follow-up care, received information on the nature of the disease and on its management, as well as they were supplied with a medication (icatibant) appropriate for the acute treatment of the next HAE attack. However, neither the patient nor her mutation-carrier son has experienced an angioedematous episode during the 6-month follow-up since establishing the diagnosis.

## 4. Discussion

To date, only a few case series of patients with PLG-HAE have been reported [3,4,13,14,15,16] and this was the first family diagnosed with PLG-HAE in Hungary.

The exact pathomechanism of PLG-HAE is yet unknown. The variant *PLG* gene leads to an amino acid change in the kringle 3 domain of plasminogen, and this results in enhanced activation of the fibrinolytic system, the generation of plasmin, and activation of the kinin-kallikrein system with consequent bradykinin release. The latter vasoactive mediator is possibly responsible for the occurrence of AE in PLG-HAE. An apparent, indirect confirmation of this is that the AE attacks occurring in PLG-HAE respond to treatment with the bradykinin B2-receptor antagonist icatibant [9,16]. Furthermore, as discussed by Napolitano et al., plasminogen harboring the K330E mutation is more prone to activation by plasminogen activators (such as tissue-type plasminogen activator (tPA) and urokinase-type plasminogen activator (uPA)), subsequently leading to the increased plasmin-mediated generation of bradykinin, the main vasoactive mediator of edematous swellings [11]. It is also noteworthy that some specific antifibrinolytic agents (such as tranexamic acid and epsilon-aminocaproic acid) reduce the severity and occurrence of AE attacks, as they bind to specific lysine binding sites in plasminogen’s kringle domain, thus decreasing the quantity of generated plasmin and regulating its downstream effects [11].

PLG-HAE is characterized by clinical manifestations that first occur usually during adulthood, most commonly involve the tongue and the face, and the acute episodes of AE may be triggered by ACEI therapy. The disorder follows an autosomal dominant trait; however, its penetrance is lower than that of C1-INH-HAE [3,4,13,14,15,16]. In HAE, genetic testing is becoming ever more important along with complement profiling. The introduction of new techniques into genetic testing (such as whole-genome sequencing) has increased the number of genes identified. In the last three years alone, five genes (*ANGPT1, PLG*, *KNG1, MYOF,* and *HS3ST6*) have been shown to have a role in nC1-INH-HAE and, as a result, fewer patients would be assigned to the U-HAE group. As shown by this study, establishing a biorepository is essential. In particular, this creates the means for the ex-post analysis of newly identified genes in stored DNA samples of the patients and, thereby, it makes the diagnosis more accurate with the possibility of subsequent family screening and the introduction of appropriate therapy.

## Data Availability

The datasets used and/or analyzed during the current study are available from the corresponding author on reasonable request.
